# Is useful the visual estimate of transmurality of hipoperfusión in stress cardiac magnetic resonance?

**DOI:** 10.1186/1532-429X-13-S1-P109

**Published:** 2011-02-02

**Authors:** Begoña M Igual, Sanchez G Juan Miguel, Buendia S Francisco, Lopez Lereu Pilar, Monmeneu M JV, lEJ Estornell, Maceira G Alicia

**Affiliations:** 1 Eresa, Valencia, Spain; 2 Hospital La Fe ., VALENCIA, Spain; 3 Hospital la Fe, VALENCIA, Spain

## Introduction

With dipyridamol stress cardiac magnetic resonance imaging (DSCMR), we obtain high spatial resolution images that allow us to assess the transmurality of hypoperfusion but the clinical usefulness of this information has not been evaluated.

## Purpose

We aimed to asses the usefulness of visual estimate of transmurality in relation to the coronary tree

## Methods

We reviewed the CMR data base( 2008-2009) to obtain data from patients with positive DSCMR and medical records to know the coronary tree. We visually asses transmurality of hypoperfusion in each of the 17 segments in all patients studied and hypoperfusion was classified as> or <50% of the segment area.

## Results

We studied, 112 consecutive patients 108 of them had performed. coronary angiography, 66 males(61%), Mean age 66±10 years 5 (4.6%) without significant coronary lesions (PPV: 95%). Patients with hypoperfusion > 50% in any segment (79 patients 73%) had a number significantly higher of affected vessels (2±0,8 vs 1,6± 0,9 p=0.04), greater number of segments with systolic dysfunction induced (SDI)((3,6±2,3 vs 0,06±0,3 p<0.001) and greater number of territories affected 1,7±0,7 vs 1,4±0,6 p=0.04). The number of segments with hypoperfusion> 50% was significantly higher in patients with SDI ( 5.9± 2,4 vs 2,4±3 p= 0.02)

## Conclusions

1.the presence of hypoperfusión > 50% indicates greater severity of coronary artery disease (CAD) with more vessels and territories afected.2.The number of segments with severe hypoperfusion is higher in patients with systolic dysfunction induced. 3. The transmurality of hypoperfusion is an indicator of severity of CAD and should be reported routinely

